# Object Perceptibility Prediction for Transmission Load Reduction in Vehicle-Infrastructure Cooperative Perception †

**DOI:** 10.3390/s22114138

**Published:** 2022-05-30

**Authors:** Pin Lv, Jinlei Han, Yuebin He, Jia Xu, Taoshen Li

**Affiliations:** 1 School of Computer, Electronics and Information, Guangxi University, Nanning 530004, China; 2013391017@st.gxu.edu.cn (J.H.); 1913301011@st.gxu.edu.cn (Y.H.); tshli@gxu.edu.cn (T.L.); 2 Guangxi Key Laboratory of Multimedia Communications and Network Technology, Nanning 530004, China; 3 China-ASEAN International Join Laboratory of Integrated Transport, Nanning University, Nanning 541699, China

**Keywords:** connected and autonomous vehicle, environment perception, transmission load reduction, object perceptibility, machine learning

## Abstract

Vehicle-infrastructure cooperative perception is an ingenious way to eliminate environmental perception blind areas of connected and autonomous vehicles (CAVs). However, if the infrastructure transmits all environmental information to the nearby CAVs, the transmission load is so heavy that it causes a waste of network resources, such as time and bandwidth, because parts of the information are redundant for the CAVs. It is an efficient manner for the infrastructure to merely transmit the information about objects which cannot be perceived by the CAVs. Therefore, the infrastructure needs to predict whether an object is perceptible for a CAV. In this paper, a machine-leaning-based model is established to settle this problem, and a data filter is also designed to enhance the prediction accuracy in various scenarios. Based on the proposed model, the infrastructure transmits the environmental information selectively, which significantly reduces the transmission load. The experiments prove that the prediction accuracy of the model achieves up to 95%, and the transmission load is reduced by 55%.

## 1. Introduction

In recent years, connected and autonomous vehicles (CAVs) have attracted much attention from both academic and industrial circles. CAVs are expected to become mainstream in future transportation systems, which improve traffic safety and efficiency [1,2].

**Figure 1:**
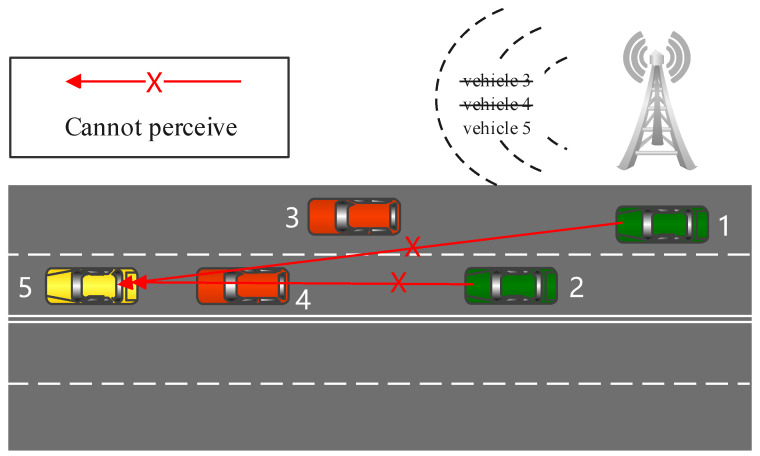
Scenario of broadcasting information selectively.

Environment perception is a critical and fundamental task for CAVs because they need to make behavioral decisions according to the environmental information. However, environment perception is prone to be influenced by obstacles and distance, and CAVs can hardly obtain complete environmental information by themselves. To solve such problems and to assist CAVs to perceive hidden vehicles and pedestrians blocked by obstacles such as buildings, a cooperative perception between vehicles and roadside infrastructure is proposed. The environment perception accuracy of CAVs can be improved significantly if roadside nodes transmit environmental information to them [3]. Nevertheless, the transmission load can easily exceed the channel capacity using such methods due to the limited bandwidth of the wireless channels [4], which imposes many limitations on CAVs. For example, in reference [5], vehicle speed is limited to ensure safe data rates for V2I (vehicle-to-infrastructure) communications. To reduce the load of environmental information transmission and to avoid the redundancy of perception information, it is a practical solution for the roadside infrastructure to transmit object information selectively. Specifically, the infrastructure only needs to broadcast messages which the autonomous vehicles cannot perceive by themselves. As demonstrated in Figure 1, Vehicles 1 and 2 are autonomous vehicles, and the rest are traditional vehicles. In traditional methods, the roadside infrastructure broadcasts information of nonautonomous vehicles (Vehicles 3, 4, and 5) to improve traffic safety. However, the roadside infrastructure can only broadcast the information of Vehicle 5 once it knows that Vehicle 5 cannot be perceived by the autonomous vehicles themselves. In this case, it is necessary for the roadside infrastructure to predict whether the objects are perceptible to the CAVs.

In [6], we propose a neural network model to predict the perceptibility of objects. Based on the model, the roadside infrastructure broadcasts the unperceived information to autonomous vehicles selectively. The model is trained and verified based on a real dataset, and its effectiveness is evaluated using IOU (intersection-over-union) as an indicator. In this paper, we further explore the factors affecting the perceptibility of objects. It is found that inaccurate predictions are more likely to be made in some special scenarios (such as crowed roads). Therefore, we propose a data filter which filters out the vehicles in such special scenarios, and their information is broadcast by the roadside infrastructure. The information of other vehicles are broadcast selectively according to the prediction results of our previously proposed perceptibility prediction model. Extensive experiments are carried out, and the results demonstrate that the newly introduced data filter further improves the accuracy of the perceptibility prediction model, while reducing the communication load.

Vehicle-infrastructure cooperative perception has been studied in recent years. One type of method is to improve the sensing capability of CAVs directly by data fusion. Duan et al. [7] propose an environment perception framework, in which CAVs fuse their own sensor data with the data of roadside infrastructure to improve sensing capability. A centralized fusion scheme is also proposed in [8], and the sensing data is sent by CAVs and fused by roadside infrastructure. Another type of method indirectly improves the perception capability by sending assistance messages according to V2I communication. For example, Rebsamen et al. [9] set up a roadside infrastructure to send messages to self-driving cars and let them slow down when pedestrians appear at the intersection. Liu et al. [10] evaluate the risk level through special roadside infrastructure; thus, autonomous vehicles can effectively avoid danger by combining their own perception information and risk level. In the studies above, the roadside infrastructure sends messages to each self-driving car rather than sending it selectively, which leads to a large amount of data redundancy and heavy communication load.

To address the problems above, various approaches have been proposed. Garlichs et al. [11] contribute a set of generation rules for the collective perception about when to generate a message and which data to include in order to reduce the network load. In [12], the information is sent selectively by considering the impact of the information on the perception accuracy and the size of the information. In reference [13], the V2I communication load is reduced by a trajectory prediction approach. Noguchi et al. [14] use V2I communication for target tracking tasks, the tracker broadcasts the handover request message to the surrounding vehicles and infrastructures before the object is lost. However, these works barely notice that in many cases only the information that CAVs cannot perceive by themselves is needed, and the perceptibility of objects is not considered in the above works.

The contributions of this paper are as follows:

- A neural network model is proposed to predict the perceptibility of objects for autonomous vehicles.
- A data filter is designed to screen out vehicles which are prone to be incorrectly predicted, and the accuracy of prediction model is improved by handling these vehicles separately.
- The model is trained and the accuracy is measured on real datasets. The effectiveness of the model is proven as the perceptual capability of CAVs is improved and the communication load is reduced.

The rest of the paper is organized as follows. The system model of this paper is introduced in Section 2, and the data preprocessing is described in Section 3. In Section 4, we propose a machine-leaning-based model to predict whether an object can be perceived by vehicles, verify the prediction accuracy, and then design a data filter to improve the model. The effectiveness of the model is evaluated in Section 5. Finally, we draw conclusion in Section 6.

## 2. System Model

**Figure 2:**
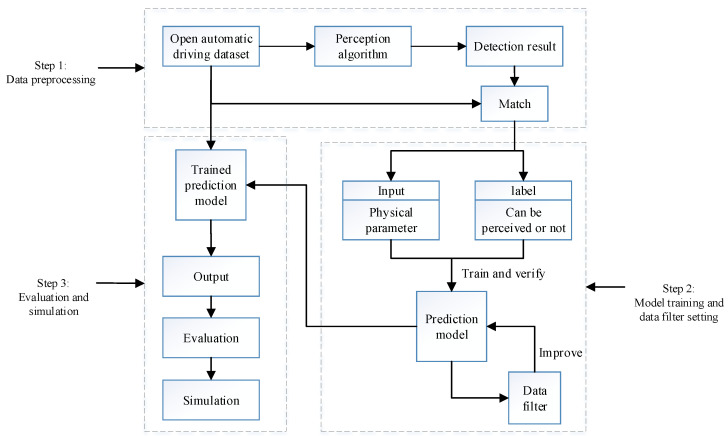
The system model. The model is built and evaluated in three steps.

As described in Figure 2. The work of this paper is carried out in three steps.

- Step 1: Data preprocessing.
  We firstly obtain the appropriate information from relevant open datasets and reconstruct a perception algorithm on the datasets. Secondly, the perception result is matched with the original data and the perceptibility (vehicle can be perceived or not) of each vehicle is recorded. Finally, the factors that may have an impact on perceptibility are screened out and processed.
- Step 2: Model training and data filter setting.
  In Step 2, a suitable perceptibility prediction model is built; it receives the processed data from Step 1 and gives a prediction of perceptibility. The model is trained with several comparative methods to verify the relationships between input factors and prediction accuracy. In addition, a data filter is proposed according to the prediction result of the model, and the data filter in turn improves the model accuracy.
- Step 3: Evaluation of the proposed model.
  In Step 3, the effectiveness of the model is evaluated. Firstly, we evaluate the effectiveness of the model in improving the perceptual capability of vehicles by the IOU (intersection-over-union) metric. Secondly, extensive simulations are conducted under several comparative methods to evaluate the effectiveness of the model regarding the reduction in the communication load.

## 3. Data Preprocessing

**Figure 3:**
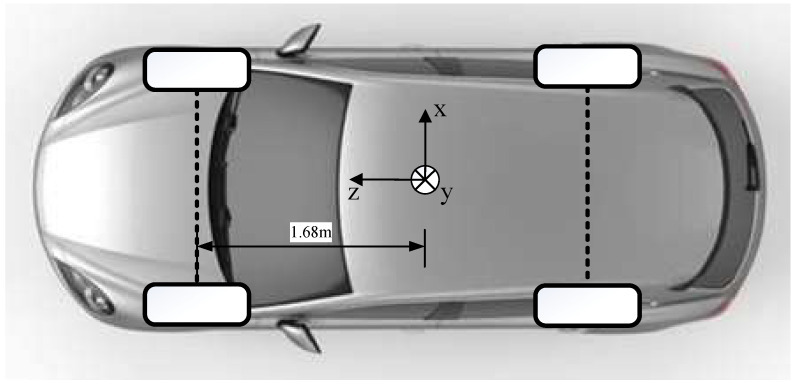
The Velodyne coordinate system of the KITII dataset. *z*-axis points in the direction of motion of the vehicle, and the *x*-axis is the direction perpendicular to it. The direction of the *y*-axis is up.

The work in this paper is based on the KITTI dataset [15]. The KITTI dataset is founded by the Karlsruhe Institute of Technology in Germany and the Toyota American Institute of Technology. This dataset is currently one of the most important public datasets in the field of autonomous driving, which is widely used in 3D object detection, visual odometry, and stereo evaluation. The KITTI dataset provides a large amount of data which is obtained from real scenes such as urban roads, villages, and highways, and the sensing data include camera pictures, lidar point cloud files, coordinate conversion information and labels of the data, etc. The labels of the data include the type of objects (car, cyclist, pedestrian, etc.), the degree of occlusion and truncation of the object, etc. The data of the KITTI dataset are based on the Velodyne coordinate, and Figure 3 shows the settings of the Velodyne coordinate.

In this work, we reconstruct an object detection algorithm PointRCNN [16], which is based on the KITTI dataset, to obtain the predicted object positions, self-rotation angles of objects, and other information. To match each object in the label of the KITTI dataset with the detection result, we control the deviation δ of the object center position on the 2D plane. Firstly, the label of each object from the KITTI dataset is traversed to record the Velodyne coordinates $x_{a}$ and $z_{a}$ of them. The object is considered to be detected if the predicted $x_{p}$ and $z_{p}$ in the predicted result satisfies formula (1).

(1) $$\begin{array}{c} \left|x_{a}-x_{p}\right|<\delta \\ \left|z_{a}-z_{p}\right|<\delta \end{array}$$

In the next step, we analyze the data in the KITTI dataset carefully and clean the data. After many attempts, we screened out six types of factors which have a great impact on the perceptibility of the objects, including the coordinate information (x,y,z), the vehicle size information (h,w,l), the degree of occlusion of the vehicle *o*, the self-rotation angle of vehicle θ, and the degree of truncation of the vehicle *t*. These factors are used as the input of the prediction model. The detail of all these factors are shown in Table 1.

Because values of the Velodyne coordinate (x,y,z) are relatively larger than the vehicle size (w,h,l), the input needed to be normalized. In this paper, the standard deviation is used as the normalization method. The normalization formula is shown as follow, where μ represents the mean and σ represents the variance.

(2) $$x_{normal}=\left(x-\mu \right)/\sigma$$

## 4. Method and Verification

In this section, a perceptibility prediction model (*PPM*) is built to predict whether the objects can be perceived, and the effects of each input factor on the prediction results are verified. In addition, a data filter is proposed to improve the performance of the *PPM*.

### 4.1. Training and Verification of the Model

**Figure 4:**
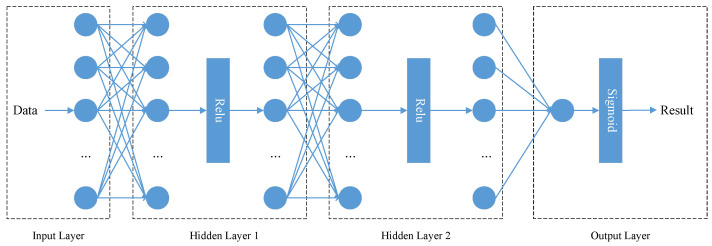
Structure of the *PPM*.

As shown in Figure 4, the *PPM* is built as a neural network, which consists of four layers. The first layer is the input layer which receives external factors such as the size of objects as the input. There are two hidden layers after the input layer. The output of each hidden layer is the input of the next layer through the Relu activation function. The output layer maps the output to “0” or “1” through the Sigmoid function, where “1” represents that the object is perceptible, and “0” denotes that the object is imperceptible. After many attempts, we find the best parameter settings for the model, and the specific parameter settings of each layer in the *PPM* are shown in Table 2.

**Figure 5:**
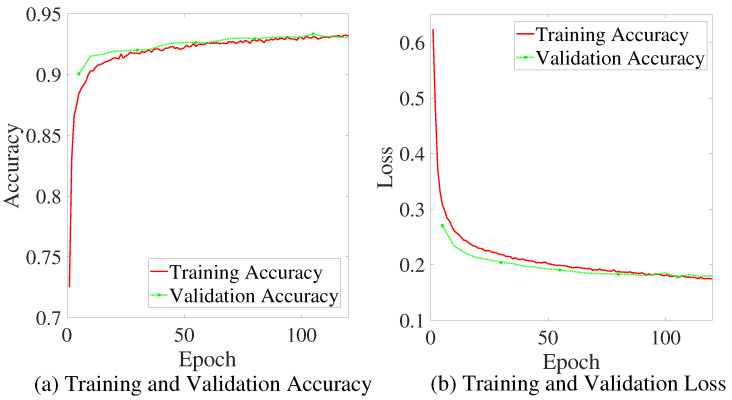
The accuracy and loss of the *PPM*.

The KITTI dataset is divided into two parts, 80% as training set and 20% as testing set. The model is trained with 120 epochs with a learning rate of 0.005; the mean-square error (MSE) is used as the loss function, and the model is verified on the testing set every 5 epochs. Prediction accuracy and the MSE loss are recorded during the training process, and they are depicted in Figure 5a,b, respectively.

The results reveal that the external factors have a great influence on the perceptibility of the objects. After training, the prediction accuracy of the testing set and training set reached 93%. It is possible to accurately predict whether an object can be detected according to these factors.

Although the factors we screen out are highly related to the perceptibility of the objects, the effect on the prediction result may vary under different factors. Therefore, we try to figure out the effect of different factors on the prediction accuracy, and the six factors below are removed from the input of the *PPM* separately to set up control groups:

1. Coordinate of object (x,y,z).
2. Size of object (w,h,l).
3. Degree of occlusion (*o*).
4. Self-rotation angle (θ).
5. Degree of truncation (*t*).
6. Set of (o,θ,t).

**Figure 6:**
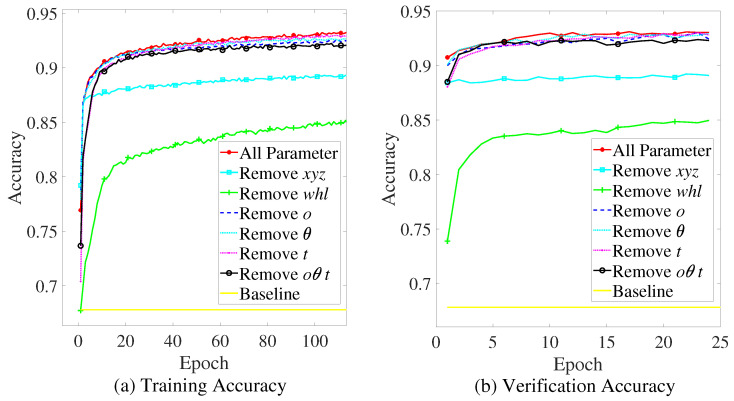
Training and validation accuracy of the control group.

The *PPM* are trained with different inputs separately, and the accuracy of prediction of the training set and the testing set are recorded, as shown in Figure 6a,b. Considering that if the proportion of positive samples is much larger (or much smaller) than negative samples, it would obtain a high accuracy rate by setting all prediction results as positive samples (or negative samples), so the proportion of positive samples is used as the control condition baseline, which is 0.678. The accuracy of the last epoch of training on the training set and the testing set are shown in Table 3.

According to the comparative experiment, the following conclusions can be drawn. First, the size information of the object has the greatest impact on the prediction result, after removing the object size information (w,h,l), the prediction accuracy reduces up to 85%. Second, the position of the object also affects the prediction result significantly; the prediction accuracy reduces to less than 90% after removing the position information (x,y,z). Furthermore, the occlusion degree, the self-rotation angle of vehicles, and the truncation degree have little effect on the prediction accuracy, but when removing these three factors at the same time, it results in a slight decrease in the prediction accuracy.

### 4.2. Setting and Evaluation of the Data Filter

Although the average prediction accuracy of the *PPM* reaches 93%, it is noted that its performance is imbalanced in different scenarios. For instance, the prediction accuracy of the *PPM* reaches 97% in most scenarios, while the prediction accuracy of it is lower than 83% in a few scenarios, which indicates an unsatisfactory performance of the *PPM* in certain situations. Therefore, we further explore the factors which affect the perceptibility of objects and find that due to the limitations of sensors and the perception algorithm, a wrong prediction is more likely to be made in some special scenarios, such as crowed roads. In this section, we try to figure out in which scenario the inaccuracy prediction would be made, and the information of all vehicles in such scenarios is broadcast; the information of vehicles not in such scenarios is broadcast selectively according to the prediction result of the *PPM*. In this way, the accuracy of the *PPM* can be improved and its performance in various scenarios can be balanced.

#### 4.2.1. Statistical Analysis

Scenarios which contain inaccuracy prediction samples are separated from the KITTI dataset and named the vulnerable dataset, and the rest of the dataset is named the stable dataset. To determine the difference between these two datasets, we count the number of vehicles separately, and the statistical data is shown in Table 4. It is obvious that the number of vehicles in the vulnerable dataset is significantly larger than the stable dataset.

**Figure 7:**
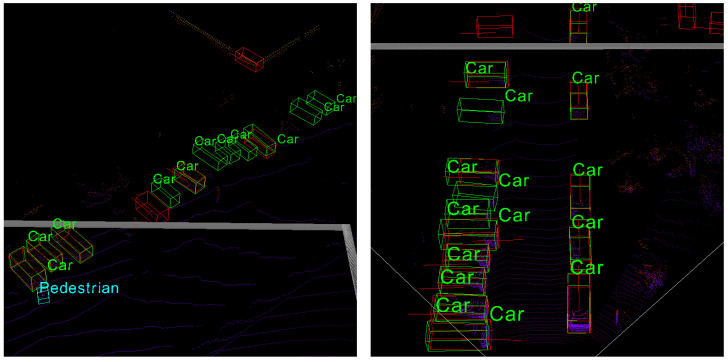
Scenarios of multiple vehicles. The vehicles approach each other, and such scenarios often contain incorrect prediction samples.

In addition, we visualize the point cloud and find that the scenarios of multiple vehicles approaching each other are relatively common in the vulnerable dataset, as illustrated in Figure 7. Therefore, a data filter can be built to separate the scenarios which are prone to result in an inaccurate prediction due to the distinctions of the vulnerable dataset.

#### 4.2.2. Data Filter Setting and Evaluation

**Figure 8:**
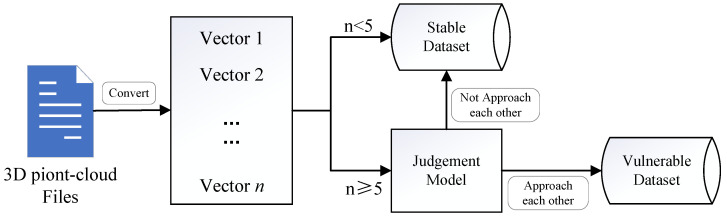
Structure of the data filter.

The structure of the data filter is sketched in Figure 8. Firstly, each scenario is converted to a tensor with *n* dimensions, where *n* is equal to the number of vehicles it contains. Secondly, the scenarios in which n<5 are divided into the stable dataset. Then, a judgment model is built to judge whether vehicles are approaching each other in a scenario, and the rest data are divided into the vulnerable dataset or the stable dataset according to the judgment model. As for the judgement model, a boundary threshold and an area threshold are set to determine whether a scenario such as the one in Figure 7 appears.

After screening out the vulnerable dataset, the information of vehicles in it is broadcast by roadside infrastructure, and the prediction accuracy of preceptibility of the rest dataset can be improved. Ideally, most of the vulnerable data and few other data are filtered out, and the preceptibility prediction accuracy reaches a high value. Therefore, we set three metrics to evaluate the data filter:

1. $p_{1}$: Means the filtered-out vulnerable data as a percentage of the total vulnerable data.
2. $p_{2}$: Means the filtered-out stable data as a percentage of the total stable data.
3. acc: Means the accuracy of the preceptibility prediction model after filtering out some data.

We except a large value of $p_{1}$ and a small value of $p_{2}$. However, these two metrics have a positive correlation, hence, we set some restrictions and adjust the threshold parameter of the judgement mode to obtain the best performance of each metric, as listed in Table 5. The parameter $fix\_p_{1}$ represents the minimum value of $p_{2}$, while $p_{1}$ is larger than 0.5; $fix\_p_{2}$ means the maximum value of $p_{1}$, while $p_{2}$ is less than 0.2, and Max_acc means the global maximum accuracy of the preceptibility prediction model. The prediction accuracy can reach more than 0.95 in all three situations above.

**Figure 9:**
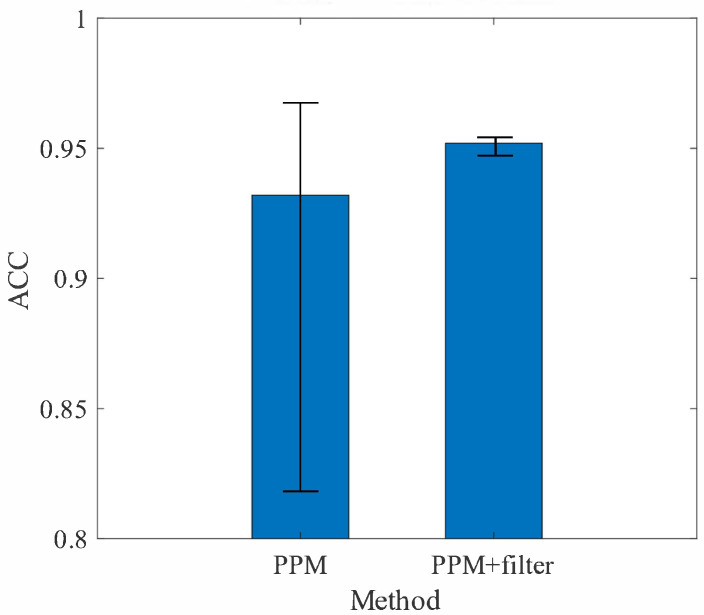
Prediction accuracy of the *PPM*. The bars on the histogram indicate the range of prediction accuracy in different scenarios.

In addiction, experiments are conducted to evaluate the effectiveness of the data filter in improving the accuracy of the *PPM* in the worst case. Traffic scenarios in the KITTI dataset are randomly used as the input and of the *PPM*, and the prediction accuracy of not adding the data filter and adding the data filter are recorded and shown in Figure 9.

The experimental results clearly show that the addition of the data filter not only improves the prediction accuracy of the *PPM* but also effectively improves the performance of the *PPM* in the worst case. Before adding the data filter, the average prediction accuracy of the *PPM* is approximately 93%, and the best performance of prediction accuracy is up to 97%, while in the worst case the prediction accuracy is only approximately 83%. After adding the data filter, the average prediction accuracy of the *PPM* is approximately 95%, and the variations of prediction accuracy are less than 1% in various scenarios.

## 5. Evaluation of the *PPM*

In Section 4, it was verified that the *PPM* had a high accuracy in the perceptibility prediction of an object, and cooperative messages can be broadcast selectively based on the prediction result. Specifically, the information of an object is broadcast if it is predicted to be imperceptible for CAVs; on the contrary, the information of an object is not broadcast if it is predicted to be perceptible for CAVs. This approach effectively reduces the communication load; however, the reduction in the broadcast cooperative messages may result in a decline in the perceptual capability of CAVs at the same time. In this section, we firstly evaluate the effectiveness of our method in improving the perceptual capability of CAVs. Moreover, a simulation experiment is conducted to verify the reduction in the communication load.

### 5.1. Effectiveness in Improving Perceptual Capability

**Figure 10:**
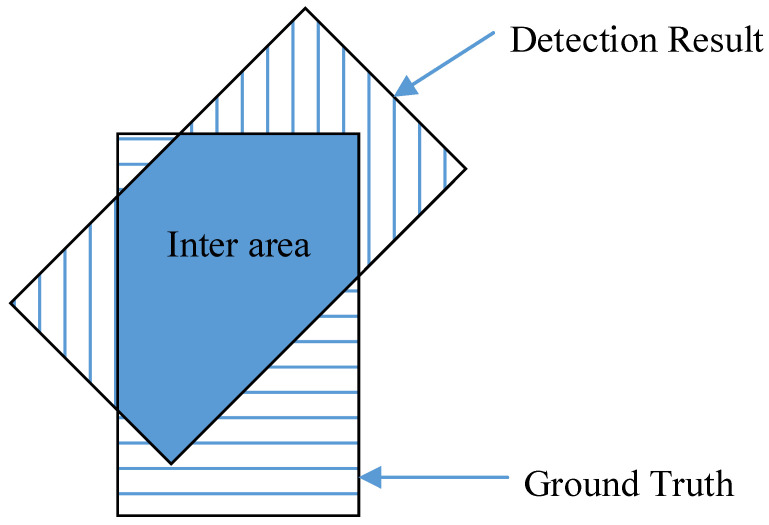
Area definition in the IOU calculation.

For different CAVs, the effectiveness of cooperative messages in improving the perceptual capability is different. For example, an object may be perceived precisely by some CAVs, but it may not be perceived by other CAVs. For the CAVs that can perceive the object, the cooperative perception messages are not necessary. On the contrary, for the CAVs that cannot perceive the object, the cooperative perception messages are useful for improving their perceptual capability. Therefore, to evaluate the effectiveness of a cooperative perception message about an object, it is essential to estimate the perceived accuracy of the object. In this section, the intersection-over-union (IOU) [17] is used as a metric to describe the perceived accuracy of objects. The IOU is demonstrated in Figure 10, the occupied area of the object in the actual label of the dataset is marked as *GroundTruth*, the occupied area of the predicted object position is marked as *DetectionResult*, and the occupied area of the intersection area is marked as *InterArea*. The IOU calculation formula is as follows:

(3) $$IOU=\frac{InterArea}{DetectionResult+GroundTruth-InterArea}$$

**Figure 11:**
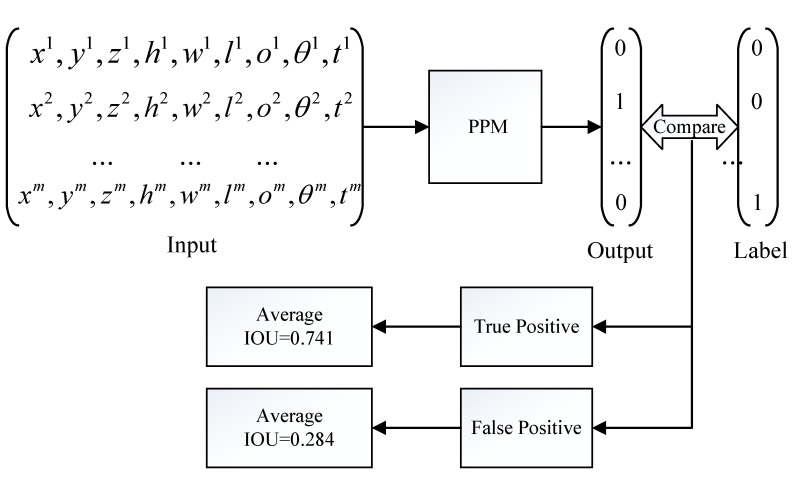
The process of evaluation. Since the negative samples have no IOU value, only the IOU of positive samples are counted.

We use all the data (including the training set and testing set) as the input of the *PPM* and obtain the prediction result of whether each object can be detected, then compare it with the actual label of the KITTI dataset and obtain two sets of True Positive (TP) and False Positive (FP). Finally, we calculate the average IOU of the object detection in the two sets separately. The process of evaluation is sketched in Figure 11.

TP sets include detected objects which are predicted as positive samples by the *PPM*. On the contrary, FP sets include undetected objects which are predicted as positive samples by the *PPM*. According to the calculation result, the average IOU value of the FP set is 0.284, while the average IOU value of the TP set is 0.741. Two conclusions can be drawn according to the result above:

1. In our method, the information of the objects in the TP set is not broadcast by the roadside infrastructure because they can be perceived by CAVs according to the prediction results of the PPM. Such a strategy does not reduce the perceptual capability of the CAVs because the objects in the TP set are perceived with high IOU accuracy, which means that CAVs are able to accurately perceive these objects by themselves, and additional cooperative messages are not helpful.
2. In our method, the information of the objects that are predicted to be imperceptible are broadcast by the roadside infrastructure. These objects can be divided into two types. In the first type, the objects are indeed not perceived by CAVs, and it is obvious that the additional cooperative messages about them are helpful in improving the perceptual capability of CAVs. In the second type, the objects can be perceived by CAVs (although they are predicted to be imperceptible); these objects belong to the FP set, and they are perceived with low IOU accuracy. Therefore, the cooperative perception messages about them are also useful in improving the perceptual capability of CAVs.

In summary, our method is effective in improving the perceptual capability of CAVs while the communication load can also be reduced.

### 5.2. Effectiveness in the Reduction in the Communication Load

#### 5.2.1. Experimental Settings

In this section, we evaluate the effectiveness of the PPM in the reduction in the communication load by conducting simulation experiments. The simulations are based on the SUMO [18] traffic simulator, and the simulation scenario is depicted in Figure 1 in Section 1. The road has four lanes, and vehicles appear randomly at the starting points of the lanes at each simulation step. At the beginning, there are no vehicles on the road. The number of vehicles gradually increases to the maximum road capacity as the experiment proceeds.

In the simulations, the cooperative perception messages are broadcast according to the following three strategies:

- All: In this strategy, the information of all vehicles in the coverage area of infrastructure is broadcast.
- PPM: In this method, the information of the vehicles which are predicted as an imperceptible object according to the *PPM* is broadcast.
- PPM + filter: In this method, the information of vehicles which are divided into the vulnerable dataset or are predicted as an imperceptible object according to the *PPM* is broadcast.

In addition, we use the traffic of the cooperative perception message (CPM) as a metric to evaluate the reduction in communication load. According to the latest standards developed by the European Telecommunications Standards Institute (ESTI) [19], CPM can be composed of multiple message containers. The cooperative message is contained in the perceived object container (POC), which contains 128 fields of the perceptual information about the object (including ID, distance, speed and acceleration, etc.). In the simulation, the information about each vehicle is contained in a single CPM, and it is broadcast by the roadside infrastructure.

#### 5.2.2. Experimental Results

**Figure 12:**
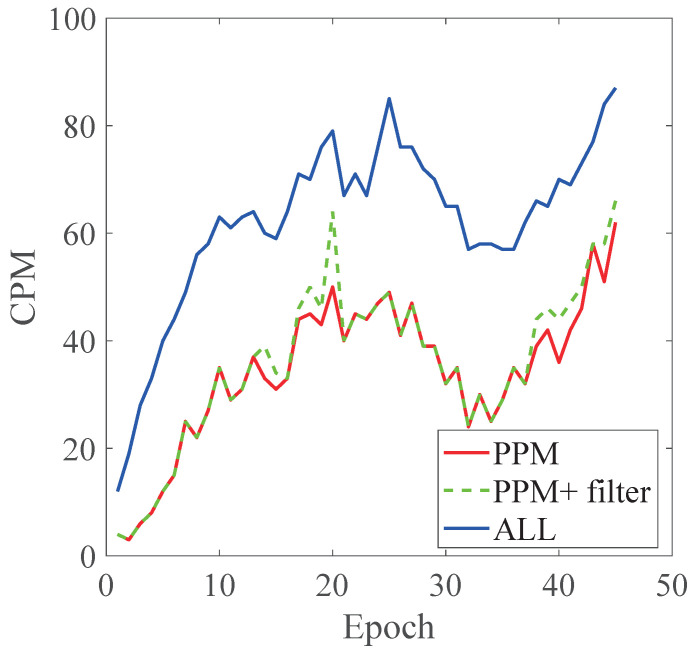
Result of simulation.

In Figure 12, the average numbers of CPMs broadcast in different methods are shown. In the All method, information about all the vehicles is broadcast by the roadside infrastructure. Therefore, the number of broadcast CPMs in the All method is equal to the number of vehicles in the road. For the PPM method, CPMs are broadcast selectively based on the prediction results of the *PPM*; thus, the average number of CPMs is significantly reduced compared with the All method, and the reduction ratio of CPMs is approximately 55%. For the PPM+filter method, the reduction ratio of CPMs is approximately 45% compared with the All method. The number of broadcast CPMs in the PPM+filter method is generally the same as those in the PPM method. However, the curves of broadcast CPMs in Figure 12 indicate that the broadcast CPMs of the PPM+filter method are still larger than those in the PPM method when the number of vehicles on the road is more than 60 and increasing (such as Epochs 10–20 and Epochs 35–45 in Figure 12). Under this condition, traffic is crowded, and more vehicles are about to enter the road; congestion such as that shown in Figure 7 can easily occur. The filter works under this condition, and it leads to an increase in the broadcast CPMs.

It is worth noting that although the performance of the PPM+filter method in reducing the communication load is weaker than that of the PPM method, as discussed in Section 4.2.2, it is better at improving the perceptual capability of CAVs. In addition, compared with the the PPM method, the PPM+filter method consumes more computational resources due to the introduction of the data filter. Therefore, the balance of the communication load reduction and the improvement of perception capability and computational resources consumption should be considered in the practical applications. For example, the PPM method can be deployed when the reduction in the communication load is the top priority. The PPM+filter method is a better choice when improving the perceived capability of CAVs is more important. Additionally, the PPM+filter method will be given lower priority than the PPM method when computational resources are insufficient.

## 6. Conclusions

In this paper, we built a perceptibility prediction model (*PPM*) to predict whether an object can be detected by an autonomous vehicle. It was proved through experiments that the model had high accuracy. In addition, a data filter was proposed to improve the accuracy of the model by consuming more computational resources. When the roadside infrastructure predicted that the autonomous vehicle cannot perceive an object according to the model, it sent the object information to the vehicles, which improved the perception range of the autonomous vehicle and reduced the communication load of roadside infrastructure. Finally, the effectiveness of the model was proved by extensive experiments.

## Figures and Tables

**Table 1:** The detail of input factors.

Factor	Physical Meaning	Range
x,y,z	The coordinates of the object	[0, 100]
w,h,l	The width, height, and length of object	[0, 20]
*o*	The degree of occlusion	{0, 1, 2, 3}
θ	Self-rotation angle of object	[−π,π]
*t*	The degree of truncation	{0, 1}

**Table 2:** Parameters of the *PPM*.

Layer	Parameters	Activate Function
Input Layer	neurons cell: 9	None
Hidden Layer 1	neurons cell: 64	Relu
Hidden Layer 2	neurons cell: 32	Relu
Output Layer	neurons cell: 1	Sigmoid

**Table 3:** Training and validation accuracy of the control group in the final epoch.

Parameter	All	Remove (x,y,z)	Remove (w,h,l)	Remove *o*	Remove θ	Remove *t*	Remove (o,θ,t)	Baseline
acc	0.932	0.894	0.853	0.926	0.928	0.928	0.922	0.678
val_acc	0.930	0.894	0.850	0.928	0.927	0.929	0.926	0.678

**Table 4:** Multiple vehicles’ proportion in different datasets.

Number of Vehicles	All	Vulnerable	Stable
≥5	0.531	0.773	0.477
≥6	0.423	0.656	0.372
≥7	0.344	0.569	0.293
≥8	0.270	0.504	0.218
≥9	0.197	0.411	0.149

**Table 5:** Best performance of the metrics.

Metric	$\mathrm{fix}\_p_{1}$	$\mathrm{fix}\_p_{2}$	Max_acc
$p_{1}$	0.501	0.401	0.657
$p_{2}$	0.265	0.194	0.378
acc	0.952	0.951	0.956

## Data Availability

The data used to support the findings of the study are available from the first author upon request.
